# Supplementary material for: Discrimination of Classical and Atypical BSE by a Distinct Immunohistochemical PrPSc Profile
Source: Pathogens. 2023 Feb 20;12(2):353. doi: 10.3390/pathogens12020353 (PMC9965285; doi:10.3390/pathogens12020353)
Supplement: Supplementary file 1 [file pathogens-12-00353-s001.zip › Sup Tables final.pdf]

Table S1: Lesion profiles in different brain regions of C-, H- and L-type BSE infected cattle (literature review)

BSE	Brainstem	Cerebellum	Midbrain	Diencephalon and Cerebrum*	Others brain regions	Remarks
C-type (field)	*Brainstem:* ++[43]<br>DMNV: +(+)[20]; +++[40,44,3]<br>*Nc. Sol. Trac., Nc. Trigem:* +++[11,16,18,19,17,20,40,44]<br>*Nc. Hyp.:* (+)[16,18,19]; +[20]<br>*Nc. Coch.:* (+)[16,18,19]<br>*Vest. Compl.:* +[16,18,19,20,40]; ++[17]; +++[3,40]<br>*Ret. Form, Olive:* +++[3,40]; +[20,44]	*Vermis:* (+)[11,16,18,19,20,43]<br>*Medulla:* +[43]<br>*Cortex:* (+)[43]	*Midbrain:* ++[43]<br>*Centr. Grey*: +++[40,11,16,17]; +(+)[19]<br>*Rostr. Coll:* ++[16,18,19]<br>*Med. Gen. Nc:* +[16,18,19]<br>*Nc. Ruber:* +++[3]; +[20]<br>*Subst. Nigra:* +(+)[20]	*Tha.:* +[41,16,18,19]; +(+)[43]; ++[40]<br>*Hyp.:* ++[11,16,18,19]; +++[40]<br>*Hip.:* (+)[43]; ++[11]<br>*Cortex:* (+)[11,16,18,19]; +[40,43]; ++[40]	*CS:* +[11]; +(+)[43]<br>*Caud.:* (+)[16,18,19]; +++[40]<br>*Put.:* +[16,18,19]; +++[40]<br>*Sept.:* (+)[16,18,19]; +[11]<br>*Claustr.:* Negative[16,18,19]<br>*OB:* (+)[43]<br>*LoP:* +[43]	Preclinical animals with mild lesions in Nc. Sol. Trac., Nc. Trigem., DMNV, Ret. Form.[41]<br>Vest. Compl. affected in early disease[54]<br>Two cows with a different lesion profile (Vermis, Tha, Cortex: B>A; MB: A>B)[40]
C-type (IC)	*Nc. Sol. Trac., Nc. Trigem:* +++[28]<br>*Nc. Hyp.:* (+)[28]<br>*Nc. Coch, Vest. Compl.:* ++[28]	*Vermis*: (+)[28]	*Centr. Grey, Rostr. Coll., Med. Gen. Nc.:* +++[28]	*Tha., Hyp.:* +++[28]<br>*Cortex:* (+)[28]	*Caud.:* +[28]<br>*Put:* +++[28]<br>*Sept.:* (+)[28]<br>*Claustr.:* (+)[28]	Lesion profile comparable to natural infected BSE cases[28]
H-type (field)	*Brainstem:* +[23]<br>*Nc. Sol. Trac., Nc. Trigem:* +++[21]; +[22]<br>*DMNV:* less severe[21]<br>*Olive:* +++[21]	NA	*Midbrian:* +[23]	NA	NA	No amyloid in brainstem[22]
H-type (IC)	*Brainstem:* ++(+)[27]<br>*Nc. Sol. Trac., Nc. Trigem:* +++[26]<br>*Nc. Hyp.:* +[26]<br>*Nc. Coch, Vest. Compl.:* ++[26]	*Vermis:* ++[26]; ++(+)[27]	*Centr. Grey, Rostr. Coll.:* +++[26]<br>*Med. Gen. Nc.:* ++(+)[26]	*Tha.:* ++(+)[27]; +++[26]<br>*Hyp.:* +++[26]<br>*Hip.:* +++[27]<br>*Cortex:* ++[25,26,30,31,29],; ++(+)[27]	*Caud.:* ++[26]<br>*Put.:* ++[26]<br>*Sept.:* +(+)[26]<br>*Claustr.:* +[26]<br>*BG:* ++(+)[27]<br>*LoP:* +++[27]	Brainstem with mildest lesions in pons and Nc. Hyp. [27]<br>C-Type with no lesion in Vermis[26], with less lesions in Cortex[30,31,26], Caud. and Put[26], but more lesions in Sept. [26]

L-type (field)	NA	NA	NA	*Tha.:* +[7<br>*Hip., Cortex:* negative[7	NA	Brainstem lesions are not consistently seen[7]
L-type (IC)	*Nc. Sol. Trac., Nc. Trigem.:* +++[24,25,29]<br>*Nc. Hyp.:* Negative[24]; (+)[25,29]<br>*Nc. Coch. Vest. Compl.:* (+)[24]++[25,29]	*Vermis:* (+)[24]; +[25,29]	*Centr. Grey, Rostr. Coll.:* +++[25,24,29]<br>*Med. Gen. Nc.:* ++[24]; +++[25,29]	*Tha., Hyp:* ++[24,25,29]<br>*Cortex:* (+)[6]; ++[5,10,11,14]	*Caud.:* (+)[24]+[25,29]<br>*Put.:* (+)[24]; ++[25,29]<br>*Sept., Claustr.:* (+)[24]; +[25,29]	C-type with less lesions in Cortex[30,31]<br>C-type with distinctly more lesion in Put. [24]
L-type (oral)	*Brainstem:* +[47]<br>*DMNV:* negative[47]	*Cortex:* negative[47]	NA	*Tha.:* +[47]<br>*Cortex:* negative[47]	*OB:* negative[47]	NA

Legend: Nc./Ncc. = nucleus/nuclei; DMNV = Dorsal motor nucleus of the vagus nerve; Nc. Sol. Trac. = Nucleus of the solitary tract; Nc. Trigem. = Nucleus of the spinal tract of V; Nc. Hyp. = Nucleus hypoglossus; Nc- Coch. = Nc. cochlearis; Vest. Compl. = Vestibular nuclear complex; Nc. Cun = Nucleus cuneatus; Olive = olivary nuclei; Ret. Form.: reticular formation; Centr. Grey = Central Grey matter; Rostr. Coll. = Rostral Colliculus; Med. Gen. Nc. = Medial geniculate nucleus; Tha. = Thalamus; Hyp. = Hypothalamus; Hip. = Hippocampus; BG = Basal ganglia; CS = Corpus striatum; Caud. = Nc. caudatus; Put = Putamen; Sept. = Septal nuclei; Claustr. = Claustrum; OB = Olfactory bulb; LoP = Lobus piriformis; (+) = weak, + = mild, ++ = moderate, +++ = severe lesions; NA = not available; *Frontal Cortex[11,16,18,19,24,25,28,29,30,31,43], or cerebral Cortex[7,27,40,47]; references are to the main text.

Table S2: PrP^{Sc} profiles in brainstem described in literature for C-, H- and L-type BSE infected cattle (literature review)

BSE	Brainstem						
	**DMNV**	**Nc. Sol. Trac.**	**Nc. Trigem.**	**Olive**	**Ret. Form**	**Brainstem in general**	**Others/Remarks**
C-type (field)	*PrP^{Sc}: +++*[45]<br>PART[40,42],<br>F-C-PART+++[44],<br>C-PART-CoL45],<br>CoL+[44]<br>ITMG[42,45]+ [44],<br>STEL++[44]<br>ITNR[40]+ [44], less ITNR[45]<br>PNER[40]+ [44],<br>LIN+[44]	*PrP^{Sc}: +++*[45]<br>PART[40,42,19],<br>Agg.[19],<br>F-C-PART+++[44],<br>C-PART-CoL[45],<br>CoL+[44],<br>ITMG[45,19]+ [44],<br>STEL++[44]<br>ITNR[45,19]+ [44],<br>PNER+[44]<br>LIN+[44]	*PrP^{Sc}: +++*[45]<br>PART[40,42,19],<br>C-PART-CoL[45],<br>ITMG[19,45]<br>ITNR[19,45],<br>Agg. [19]	*PrP^{Sc}: +++*[45]<br>PART+++[44],<br>F-C-PART+[44],<br>C-PART-CoL[45]<br>ITMG[45]+ [44],<br>ITNR[40,45]++ [44]<br>LIN+[44]	*PrP^{Sc}: +++*[45]<br>PART[42]++ [44],<br>C-PART-CoL[45],<br>C-PART+44]<br>CoL42]+44],<br>ITMG[45],<br>STEL+[44],<br>ITNR[40,42,15],<br>PNER+[44],<br>LIN[42]+++ [44]	*PrP^{Sc}: +++*[7]<br>PART[7],<br>F-C-PART +++[41]<br>F-C-PART-CoL[43],<br>CoL++[41],<br>STEL[7,41],<br>ITNR[7,43]++ [41],<br>LIN[7,43]<br>PNER[41]	Brainstem mostly affected[43]<br>*Vest.Compl.:*<br>ITNR[19,40], ITMG[19],<br>STEL[19], PART[19],<br>LIN[19]<br>Nc. Hyp: STELL[42],<br>ITNR[19,40], PART[19]
C-type (IC)	*PrP^{Sc}: +*[28]	*PrP^{Sc}: +++*[28]	*PrP^{Sc}: +++*[28]	NA	NA	*PrP^{Sc}: +++*[28]<br>PART, STEL,<br>ITNR[24]	NA
H-type (field)	*PrP^{Sc}: +*[23]	*PrP^{Sc}: ++(+)*[22] or +[23]<br>F-PART[22],<br>PNER[22],<br>LIN[22]	*PrP^{Sc}: +++*[22] or +[23]<br>F-PART[22],<br>PNER[22],<br>LIN[22]	*PrP^{Sc}: +*[23]<br>ITNR22]	ITNR[22]	F-C-PART[22,23],<br>Agg. [8],<br>ITNR/ITMG[8,23],<br>selected Ncc.:<br>ITNR[22], PNER+[22]<br>LIN(+)[22]	*Nc. Cun.:*<br>ITNR[22] +[23]<br>*Nc. Hyp:*<br>PrP^{Sc} +[23]
H-type (IC)	No ITNR[27]<br>ITMG[61]	ITMG[61]	ITMG[61]	NA	NA	*PrP^{Sc}: +++*[27,30,26]<br>F-C-PART[31,26],<br>CoL[27],<br>ITMG[8,10,61],<br>ITNR[26,27],<br>PNER[26],<br>LIN[26]	Distinct ITMG/STEL[31] or ITMG[61]<br>*WM:* ITMG[26]<br>No ITNR with SAF32 and B103 (N-terminal) [26]

L-type (field)	*PrP^{Sc}*: (+)[8]<br>Negative[7]<br>Agg. [42]	Agg. [42]	*PrP^{Sc}*: (+)[8]<br>Agg. [42]	NA	Agg. [42]	*PrP^{Sc}*: (+)[7]<br>No PL[46]	ITNR/ExNTR[8]<br>Similar to C-Type[46],<br>consistent[30] with [46] (L-type IC)
L-type (IC)	ITNR[61]	ITNR[61]	ITNR[61]	NA	NA	*PrP^{Sc}*: +++[29,30]<br>F-C-PART[29,30],<br>CoL[30]<br>ITNR[14,61],<br>PNER[12]	Similar to C-Type[25]<br>PNER prominent[31]
L-type (oral)	*PrP^{Sc}*: +[47]	NA	NA	NA	NA	*PrP^{Sc}*: +++[47]<br>F-C-PART[47],<br>*Nc. Cun:* PNER[47]	No PL[47]

*Legend***:** DMNV = Dorsal motor nucleus of the vagus nerve; Nc. Sol. Trac. = Nucleus of the solitary tract; Nc. Trigem. = Nucleus of the spinal tract of V; Nc. Hyp. = Nucleus hypoglossus; Vest. Compl. = Vestibular nuclear complex; Nc. Cun = Nucleus cuneatus; Olive = olivary nuclei; Ret. Form.: reticular formation; Nc./Ncc. = nucleus/nuclei; PrP^{Sc} = quantity of PrP^{Sc}; (+) = weak, + = mild, ++ = moderate, +++ = severe accumulation of PrP^{Sc}; PART = PrP^{Sc} deposition on cell surface in different appearance: F-G-PART-CoL = fine (F) and/or coarse (C) PART accumulation up to a coalescing (CoL) pattern; ITMG = intramicroglial; STEL = stellate; ITNR = intraneuronal; PNER = perineuronal; LIN = linear; Agg. = aggregates; PL = plaques; NA = not available; anti-PrP antibodies used recognize the core region of the protein: F99[7,8,22,23,24,26,27,29,42,47]; R145[19,30,31,46]; 6H4[25,41,43]; T1[28,45];12F10[8]; C15S[44]; L42[43]; SAF84[40]; F99/6C2[61]; references are to the main text.

Table S3: PrP^{Sc} profiles in different brain regions described in literature for C-, H- and L-type BSE infected cattle (literature review)

BSE	Cerebellum	Midbrain	Thalamus	Hypothalamus and Hippocampus	Cerebrum	Other brain regions	Remarks
C-type (field)	*Cortex:* F-C-PART+[41]; ITNR(+)[41]; PNER[41,43]; STEL++[41,43]<br>*MLay:* STEL++[45]<br>*Medulla:* PrP^{Sc}++[45], F-C-PART[43,45]; ITNR[43,45]; STEL[43]; LIN[43,45]<br>*Vermis:* STEL[43]; F-C-PART[43]	*PrP^{Sc}:* +++[25,45]<br>PART[25], F-C-PART-CoL[43,45], PART-CoL++[41], STEL[43]++[41], ITNR[41,43,45](Red Nc.[40])<br>PNER[41,43,]<br>LIN[41,43], PL[25]<br>*Subst. nigra:* PART[40]<br>*Centr. Grey:* PL[40] (not in[19]), ITNR[19], STEL[19], PART[19], Agg[19]	*PrP^{Sc}:* +++[7,45]<br>PART[7,19,40,43], F-C-PART-CoL[43,45], PART-CoL++[41]<br>STEL[7,19,43,45],+[41], ITNR[7,19,40,43,45]++[41], PNER[45]++[41], LIN[7,19,43,45]++[41], PL[40], Agg. [19]	*Hyp:* PrP^{Sc} +++[45]<br>ITNR[19,40,45], F-C-PART-CoL[45], Agg. [19], STEL[19,45]<br>PNER[19,45]++41], LIN[19,45]<br>*Hip:* PrP^{Sc} ++[45]<br>F-C-PART in GLay[45], STEL[41,43] in Str. Oriens / MLay[45], ITNR[41] in Str. Pyr. [45], PNER[41,43], LIN in Str. Oriens / MLay[45]	*Cortex:* PrP^{Sc} +[7,45]<br>PART[2], (+)[19,41], F-PART[45]<br>STEL[7,19,45]++ [41], ITNR[7,45](+) [19]<br>PNER[40], LIN[7,19,41,45], PL[40]	*CS:* F-C-PART[45]+41], CoL[41] STEL[41,45], ITNR[45], LIN[41,45]<br>*OB[7]*, *LoP[7]*: PrP^{Sc} + PART, STEL, ITNR, LIN<br>*Caud-Put:* ITNR[19], STEL[19], PNER[19,40], LIN[19], Agg[19]<br>*Sept.:* ITNR[19], STEL[19], PART[19], LIN[19], (+)PNER[19], (+)Agg[19]<br>*Claustr.:* STELL[19]	Cerebral Cortex the least affected[43]
C-type (IC)	*WM:* PrP^{Sc}+++[28]<br>*GLay:* +(+)[28]<br>*In general*: PART[24], STEL[24], ITNR[24]	NA	*PrP^{Sc}:* +++[28]<br>PART[24], STEL[24], ITNR[24]	NA	*Cortex:* PrP^{Sc} +(+)[28]<br>PART[24], STEL[24], ITNR[24]	*OB[24]*, *BG[24]*: PART, STEL	NA
H-type (field)	*PrP^{Sc}:* negative[23]	*PrP^{Sc}:* +[23]<br>PART, ITNR, ITMG[23]	*PrP^{Sc}:* +[23]<br>PART, ITNR, ITMG[23]	*Hip:* PrP^{Sc} negative[23]	*Cortex:* PrP^{Sc} negative[23]	*BG:* PrP^{Sc} negative[23]	NA
H-type (IC)	*Cortex:* STEL[26]<br>*M/Lay:* PrP^{Sc}(+)[27] or +[30], PART[27], multifocal[30]	*PrP^{Sc}:* PrP^{Sc}+++[27]<br>F-C-PART[26], ITMG[27] in WM[26], ITNR[27] in different Ncc.	F-C-PART[26], STEL[26]<br>ITNR[26], LIN[26], PNER[26]	*Hyp:* PrP^{Sc} +++[26]<br>F-C-PART[26], STEL[26]<br>*Hip:* PrP^{Sc} ++[27]<br>CoL[27], STEL[26],	*Cortex:* PrP^{Sc} +++[26]<br>PART[30], F-C-PART[26,27], CoL[27], STEL[26,27,30], ITMG[27], ITNR[25]	*BG[26]*: PrP^{Sc} +++<br>F-C-PART, STEL, ITNR, PNER, LIN<br>*CS:* +PL[26]	Extensive PART, but distinct ITMG/STEL[31]<br>No ITNR with SAF32 and B103 (N-terminal) [26]

	WM: negative[27],<br>STEL[30], ITMG[26],<br>PL[26]<br>*Ncc.:* PrP^{Sc}<br>positive[27]<br>*In general:* F-C-<br>PART[26]			*WM:*<br>ITMG[26], PL+[26]	rarely[27],<br>PNER[26,27], LIN[26],<br>PL[26]<br>*WM:* (+) C-<br>PART[27], ITMG[26],<br>PL[26] rarely[30]		
L-type (field)	*MLay:* PrP^{Sc}+[7]<br>PL[7]	NA	PL[7]	NA	*Cortex/WM:* PL[7]	*OB:* PL[7]	No PL[46], pattern similar to C-type[46], consistent[30] with [46] (L-type IC)
L-type (IC)	*M/GLay:*<br>PrP^{Sc}+++[30],<br>homogenous[30]<br>*Ncc.:* F-C-PART[29]<br>*In general:*<br>PNER[24]	*Centr. Grey:*<br>F-C-PART[29], PL (+)[29]	*PrP^{Sc}:* +++[25]<br>PART[25], F-C-<br>PART+++[29]<br>ITNR++[29],<br>PNER[24], PL[25];<br>(+)[29]	PNER[24]	***Cortex:*** F-C-<br>PART[29],<br>ITNR+[29], PL in deep grey layers[24,29]<br>*WM:* C-PART[30],<br>PNER[30,24],<br>PL[24,27,30](+) [29]	*BG:* PL(+)[29]	Like C-type topgraphy[25]<br>In midbrain and thalamus mostly periaquae-ductual areas affected[25]<br>Extensive PART, distinct PNER[31]
L-type (oral)	NA	NA	*PrP^{Sc}:* +++[47]<br>F-C-PART[47],<br>PNER[47]	NA	NA	NA	No PL detectable[47]

Legend: Nc./Ncc. = nucleus/nuclei; MLay = Molecular layer; GLay = Granular layer; WM = white matter; Subst. = Substantia; Centr. Grey = Central grey matter; Hyp. = Hypothalamus; Hip. = Hippocampus; Str. = Stratum; Pyr. = Stratum pyramidale; BG = Basal ganglia; CS = Corpus striatum; Caud.-Put. = Nc. caudatus-Putamen; Sept. = Septal nuclei; OB = Olfactory bulb; LoP = Lobus piriformis; Claustr.: Claustrum; PrP^{Sc} = quantity of PrP^{Sc}; (+) = weak, + = mild, ++ = moderate, +++ = severe accumulation of PrP^{Sc}; PART = PrP^{Sc} deposition on cell surface in different appearance: F-G-PART-CoL = fine (F) and/or coarse (C) PART accumulation up to a coalescing (CoL) pattern; ITMG = intramicroglial; STEL = stellate; ITNR = intraneuronal; PNER = perineuronal; LIN = linear; Agg. = aggregates; PL = plaques; NA = not available; anti-PrP antibodies used recognize the core region of the protein: F99[7,8,22,23,24,26,27,29,42,47]; R145[19,30,31,46]; 6H4[25,41,43]; T1[28,45];12F10[8]; C15S[44]; L42[43]; SAF84[40]; references are to the main text.
