# Supplementary figures and images for: Discrimination of Classical and Atypical BSE by a Distinct Immunohistochemical PrPSc Profile
Source: Pathogens. 2023 Feb 20;12(2):353. doi: 10.3390/pathogens12020353 (PMC9965285; doi:10.3390/pathogens12020353)

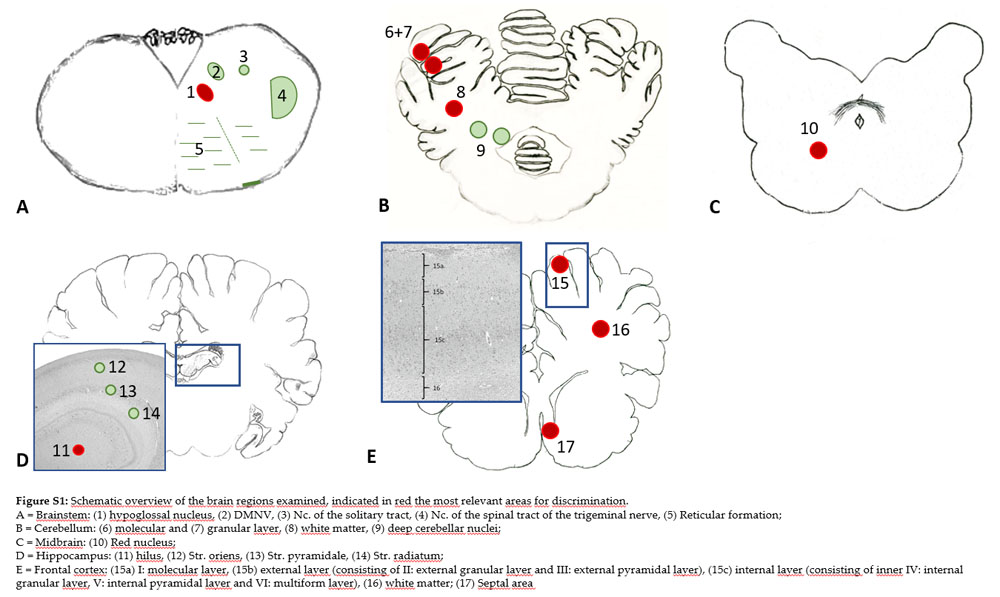

Supplement: Supplementary file 1 [file pathogens-12-00353-s001.zip › Figure S1 Neu 1000 Pixel.jpg]

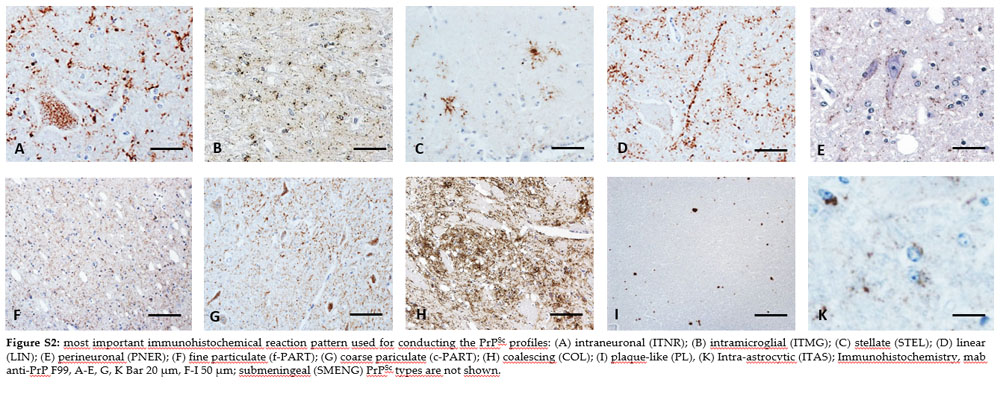

Supplement: Supplementary file 1 [file pathogens-12-00353-s001.zip › Figure S2 Neu1000 Pixel.jpg]

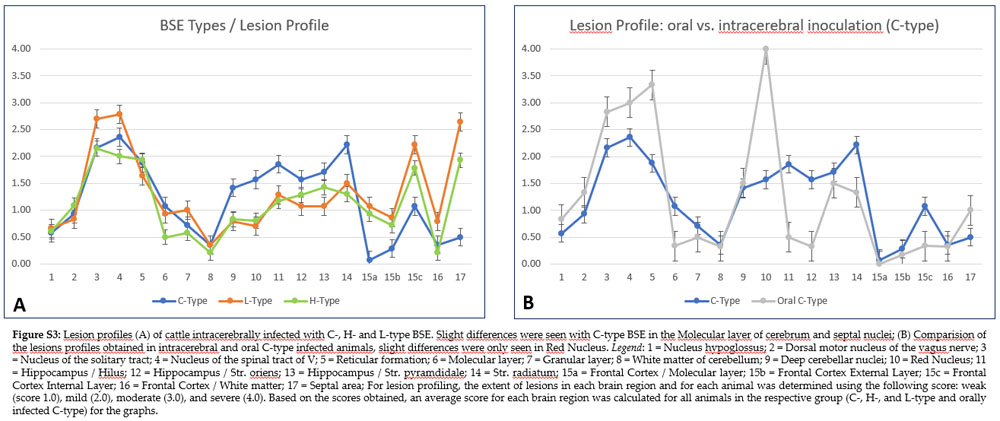

Supplement: Supplementary file 1 [file pathogens-12-00353-s001.zip › Figure S3 Neu 1000 Pixel.jpg]

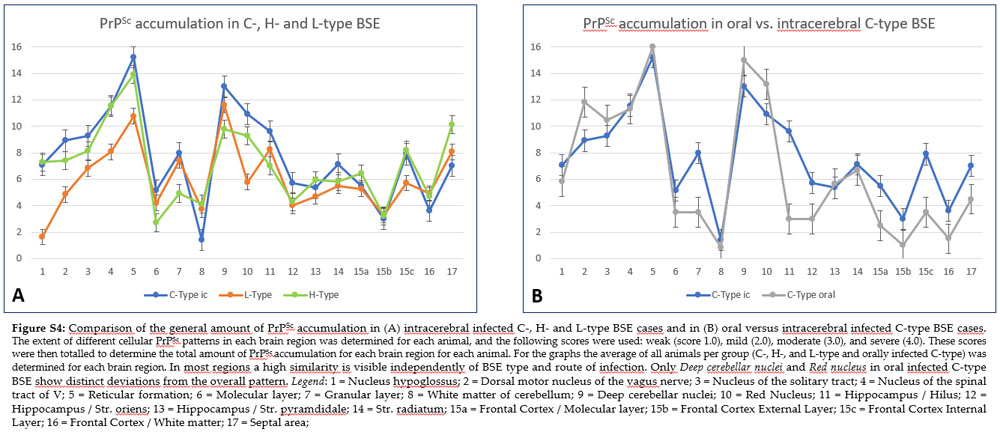

Supplement: Supplementary file 1 [file pathogens-12-00353-s001.zip › Figure S4 Neu 1000 Pixel.jpg]
